# Rosuvastatin Improves Cognitive Function of Chronic Hypertensive Rats by Attenuating White Matter Lesions and Beta-Amyloid Deposits

**DOI:** 10.1155/2020/4864017

**Published:** 2020-08-12

**Authors:** Lu Zheng, Ying Cai, Baoshan Qiu, Linfang Lan, Jing Lin, Yuhua Fan

**Affiliations:** ^1^Department of Neurology, The First Affiliated Hospital, Sun Yat-sen University; Guangdong Provincial Key Laboratory of Diagnosis and Treatment of Major Neurological Diseases, National Key Clinical Department and Key Discipline of Neurology, Guangzhou 510080, China; ^2^Department of Neurology, The Third Affiliated Hospital of Sun Yat-sen University, Guangzhou 510630, China; ^3^Department of Neurology, The Tiantan Hospital, Capital Medical University, Beijing 100070, China

## Abstract

Hypertensive white matter lesion (WML) is one of common causes of vascular cognitive impairment. In this study, we aimed to investigate the effect of rosuvastatin on cognitive impairment and its underlying mechanisms in chronic hypertensive rats. From the 8^th^ week after establishment of stroke-prone renovascular hypertensive rats (RHRSPs), rosuvastatin (10 mg/kg) or saline as a control was administrated once daily for consecutive 12 weeks by gastric gavage. Cognitive function was assessed with the Morris water maze test and novel object recognition test. WML was observed by Luxol fast blue staining. A*β* deposits, Claudin-5, Occludin, and ZO-1 were determined by immunofluorescence. After rosuvastatin treatment, the escape latencies were decreased and the time of crossing the hidden platform was increased in the Morris water maze, compared with the vehicle-treated RHRSP group. In a novel object recognition test, the recognition index in the rosuvastatin-treated RHRSP group was significantly larger than that in the vehicle-treated RHRSP group. Rosuvastatin treatment presented with the effects of lower WML grades, higher expression of tight junction proteins Claudin-5, Occludin, and ZO-1 in the corpus callosum, and less A*β* deposits in the cortex and hippocampus. The data suggested that rosuvastatin improved the cognitive function of chronic hypertensive rats partly by attenuating WML and reducing A*β* burden.

## 1. Introduction

Vascular cognitive impairment (VCI), including mild cognitive impairment of vascular origin and vascular dementia [1], becomes more popular with prolonged life expectancy [2]. White matter lesion (WML), as a major subtype of cerebral small vessel disease, is characterized by decreased attenuation on CT scans or hyperintensities on T2-weighted MRI scans in periventricular or subcortical white matter. It is a common cause of VCI [3]. Hypertension is a key factor of WML [4]. The potential mechanisms of WML include chronic hypoperfusion [5], disruption of blood-brain barrier (BBB) [6], and activation of glial cells [7]. Meanwhile, experimental studies have identified a complex correlation between neurodegeneration and A*β*3-16 [8]. A*β* deposits, as observed in VCI patients, have been proven to be significantly associated with WML [9]. They act synergistically to impair the cognitive function [10].

Statins, widely prescribed for the treatment of hypercholesterolemic patients, are known as inhibitors for the enzyme 3-hydroxy-3-methylglutaryl coenzyme A reductase. Their beneficial effects on maintaining BBB integrity have been proven in animal models of sepsis [11], multiple sclerosis [12], epilepsy [13], and stroke [14]. Thus, we proposed the hypothesis that statins in the long-term hypertension-induced WML rat model could ameliorate BBB disruption and further attenuate WML while reducing A*β* deposits, to finally improve the cognitive function.

## 2. Materials and Methods

### 2.1. Animals

The experimental protocol was approved by the ethics committee for animal research of Sun Yat-sen University, and all procedures involving animals were conducted in conformity with the ARRIVE guidelines and the National Institutes of Health Guide for the Care and Use of Laboratory Animals (NIH Publications No. 8023, revised 1978). Male Sprague-Dawley rats (*n* = 71, 4-week-old, Medical Laboratory Animal Centre in Guangdong Province, Guangzhou, Guangdong, China) were kept under a constant light-dark cycle (8:00 a.m. to 8:00 p.m. light period), at an ambient temperature of 22 ± 1°C, with free access to water and food.

### 2.2. Surgery

The stroke-prone renovascular hypertensive rat (RHRSP) model was established in 60 male Sprague-Dawley rats as described by Zeng et al. [15]. The rats were fasted overnight before the day of the experiment, with free access to water. Briefly, rats underwent a median longitudinal incision on the abdominal skin after being anesthetized with 1% pentobarbital (50 mg/kg, intraperitoneal injection). Both the right and left renal arteries were occluded by a ring-shaped silver clip (0.3 mm in diameter). Additional 11 sham-operated rats (SHAM) were used as controls. Systolic blood pressure was monitored every other week by a tail-cuff sphygmomanometer (ML866 Powerlab 4/30, AD Instruments Pty Ltd., Sydney, NSW, Australia).

### 2.3. Rosuvastatin Treatment

At postoperative week 8, 56 RHRSP rats with systolic blood pressure higher than 160 mmHg and no stroke symptom were selected for a further study. RHRSP rats were randomly divided into two groups: vehicle-treated RHRSP group (RHRSP/veh) and rosuvastatin-treated RHRSP group (RHRSP/ros). The RHRSP/ros group was treated with rosuvastatin calcium tablets daily (10 mg/kg; AstraZeneca, London, UK) by gastric gavage for 14 weeks, while the RHRSP/veh and sham-operated groups were treated with saline. At postoperative week 22, 11 sham-operated rats, 19 RHRSP/veh rats, and 19 RHRSP/ros rats underwent the next experiment. The rest 18 rats were excluded due to death.

### 2.4. Novel Object Recognition Test

At postoperative week 20, the novel object recognition test was performed in all rats [16] in a rectangular plywood open field measuring 100∗60 cm with 50 cm high walls. The floor was covered with sawdust. Double copies used in this test presented with different colors and shapes, which were made either of glass, wood, plastic, or metal. In the habituation phase, rats were left to explore the empty arena for 10 minutes per day for three days (habituation). The familiarization phase took place on the fourth day. Two identical objects (F1 and F2) were placed 10 cm away from the sides of the arena at 50 cm apart. Each rat was placed into the arena in turn and given a maximum of 10 minutes to explore the object. The retention phase took place 24 hours after familiarization. Rats were given three minutes to explore the open field where one familiar (F) and one novel (N) object were placed. Exploration was defined as the rat putting its head within 2 cm of the object while looking at, sniffing, or touching it. The objects were washed with 70% ethanol solution between trials. Scoring was noted by experimenters blind to treatment assignment. Time for exploring the novel object and the recognition index were recorded. The recognition index calculated for each rat was expressed by the ratio *N*/(*N* + *F*) (*N* = time for exploring the novel object and *F* = time for exploring the familiar object).

### 2.5. Morris Water Maze

At postoperative week 21, the water maze test was performed in all rats. The first day was the acclimatization trial when rats were allowed to explore in a pool (160 cm diameter) for three minutes without a platform. In the hidden platform acquisition test, four training trials were conducted in rats every day. During each trial, rats were placed into the maze, facing the edge, from four positions. A trial was deemed to be completed if the rat climbed onto the platform and stayed there for 10 seconds. If a rat failed to find the platform within 60 seconds, it would be guided to the platform by a stick and placed on the platform for 15 seconds. All rats received four training trials per day for five days, followed by a probe trial on the sixth day. In the probe trial, rats were placed into the maze for 60 seconds in the absence of the platform, during which the number of times of crossing the platform zone was recorded. Data were analyzed from videotape recording (Morris water maze, DMS-2; Institute of Materia Medica, Chinese Academy of Medical Sciences & Peking Union Medical College, Beijing, China).

### 2.6. Tissue Preparation

At postoperative week 22, after deep anesthesia with 1% pentobarbital, rats were perfused transcardially with 0.9% sodium chloride at 4°C followed by 4% paraformaldehyde in 0.1 M phosphate-buffered saline (PBS, pH 7.4). The brains were fixed in the same fixative for 24 h and cryoprotected in 20% and 30% sucrose in 0.1 M PBS overnight at 4°C. Coronal sections (10 *μ*m thick) between 5.2 and -4.5 mm from the bregma were obtained using a freezing microtome (Leica, Wetzlar, Hesse-Darmstadt, Germany).

### 2.7. Luxol Fast Blue Staining

Sections between 1.5 and -1.5 relative to the bregma were selected for Luxol fast blue staining to examine the nerve fibers and myelin. Serial sections were selected at intervals of every sixth sections from each rat for quantification. The sections were hydrated in 100% alcohol and then 0.1% Luxol Fast Blue solution in a 56°C oven overnight. After rinsing off excess Luxol Fast Blue with 95% alcohol and distilled water, sections were differentiated in lithium carbonate solution and 70% alcohol for 20 seconds each. Thereafter, sections were stained with eosin for 20 seconds and then dehydrated and cleared using alcohol and xylene, respectively. Images were captured under a microscope (Olympus, Tokyo, Japan). The severity of WML was graded in accordance with previous reports [7]. The degree of white matter changes was graded as normal (grade 0); disarrangement of nerve fibers (grade 1); formation of marked vacuoles (grade 2); and disappearance of myelinated fibers (grade 3).

### 2.8. Immunofluorescent Labeling

Sections between 5.2 and 4.5 mm and -2.5 and -4.5 mm relative to the bregma were selected for immunofluorescence using standard procedures. Sections were blocked with 5% normal goat serum for one hour at room temperature and then primary antibodies at 4°C overnight. The following primary antibodies were used: rabbit anti-A*β*3-16 (1 : 200, Covance, Princeton, NJ, USA), mouse anti-NeuN (1 : 500, Millipore, Bedford, MA, USA), rabbit anti-Occludin (1 : 50, Thermo Scientific, Waltham, MA, USA), rabbit anti-ZO-1 (1 : 100, Thermo Scientific), and mouse anti-Claudin-5 (1 : 80, Thermo Scientific). The secondary antibodies included anti-rabbit IgG (H+L) (Alexa Fluor 555, 1 : 1000, Cell Signaling Technology, Danvers, MA, USA), and anti-mouse IgG (H+L) (Alexa Fluor 488, 1 : 1000, Cell Signaling Technology). Images were captured under a microscope (Olympus, Tokyo, Japan) and analyzed by image analysis software (Image-Pro Plus Version 6.0, Media Cybernetics, Bethesda, MD, USA). For the quantification of immunofluorescence of Claudin-5, Occludin, and ZO-1, the number of doubled immunofluorescent vessels was counted using Image-Pro Plus image analysis software within three nonoverlapping fields (425 × 320 mm^2^) under ×400 magnification and was expressed as the average number of intact cells per field on each section. For quantification of A*β* burdens, the area of A*β*, frontal cortex, and hippocampus region were analyzed as above. Moreover, the percentage of the frontal cortex, the hippocampus region covered by A*β* immunoreactivity, was determined.

### 2.9. Statistical Analysis

Data were expressed as the mean ± standard error of mean (SEM) and analyzed using SPSS Version 20.0 (International Business Machines Corporation, Armonk, NY, USA). Systolic blood pressure and escape latency were compared by repeated-measure ANOVA. Other data were analyzed by one-way ANOVA followed by a least significant difference *t*-test. Statistical significance was defined as *P* < 0.05.

## 3. Results

### 3.1. Rosuvastatin Failed to Lower Systolic Blood Pressure in RHRSPs

**Figure 1:**
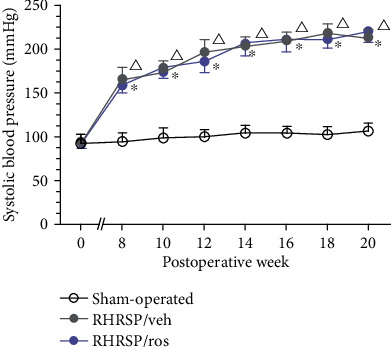
Systolic blood pressure in the experimental period. Data were presented as the mean pressure ± SEM. RHRSP/veh: RHRSP treated with vehicle; RHRSP/ros: RHRSP treated with rosuvastatin. ^∗^RHRSP/veh vs. sham-operated rats, *P* < 0.001. ^△^RHRSP/ros vs. sham-operated rats, *P* < 0.001. *n* = 19 in RHRSP/veh and RHRSP/ros; *n* = 11 in the sham-operated group.

No rat died in the sham-operated group in the experimental period. The mortality rate was 36.67% (11/30) in the vehicle-treated RHRSP group and 26.92% (7/26) in the rosuvastatin-treated RHRSP group. There was no difference in baseline systolic blood pressure among these three groups. The systolic blood pressure increased dramatically higher in RHRSPs than in the sham-operated group after the surgery. At postoperative week 20, there was no significant difference in systolic blood pressure between vehicle-treated RHRSPs and rosuvastatin-treated RHRSPs throughout the treatment (*P* = 0.059) (Figure 1).

### 3.2. Rosuvastatin Improved the Cognitive Function of RHRSPs

**Figure 2:**
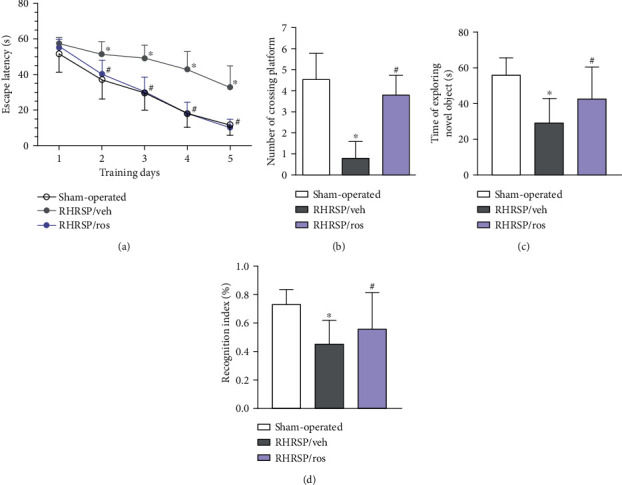
Rosuvastatin improved the cognitive function of RHRSP. (a) Escape latency in the Morris water maze. (b) The number of crossing hidden platform in the Morris water maze. (c) Time of exploring novel object in the novel object test. (d) Recognition index in the novel object test. ^∗^RHRSP/veh vs. sham-operated rats, *P* < 0.001. ^#^RHRSP/ros vs. RHRSP/veh, *P* < 0.001. *n* = 19 in RHRSP/veh and RHRSP/ros; *n* = 11 in the sham-operated group.

To assess the spatial reference learning and memory of rats, we performed the Morris water maze test. In learning trials, the escape latencies of rats in groups decreased by day with a significant difference. The RHRSP/veh group had longer latencies from day 2 to day 5 relative to sham-operated rats. Rosuvastatin treatment significantly shortened escape latencies in RHRSPs (RHRSP/ros vs. RHRSP/veh, *P* < 0.001) (Figure 2(a)). In the probe trial, the number of crossing platform was significantly decreased in the RHRSP/veh group, and rosuvastatin greatly increased the number of crossing platform of RHRSPs (RHRSP/ros vs. RHRSP/veh, *P* < 0.001) (Figure 2(b)). The trial data implied that spatial reference memory of rosuvastatin-treated RHRSPs was better improved compared with the vehicle-treated RHRSPs.

To assess the learning and memory of rats in different groups, we performed the novel object recognition test. Vehicle-treated RHRSPs showed impaired memory by reducing their exploratory capacities during the novel object test. The time for exploring the novel object and the recognition index were decreased in the RHRSP/veh group, compared with the sham-operated group. And they were significantly increased after rosuvastatin treatment (Figures 2(c) and 2(d)). The results indicated that rosuvastatin rescued the impairment of 24 h retention of novel object recognition in RHRSP independently of blood pressure.

### 3.3. Rosuvastatin Ameliorated the Severity of WML

**Figure 3:**
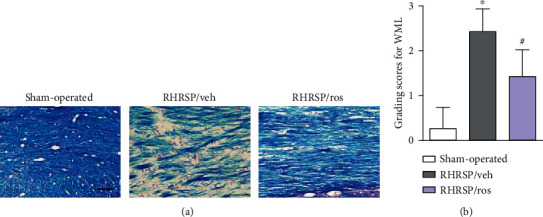
Rosuvastatin ameliorated the severity of WML. (a) Representative images of Luxol fast blue staining of the corpus callous. Scale bar: 50 *μ*m. (b) Grading scores for WML. RHRSP/veh vs. sham-operated, *P* = 0.0027; RHRSP/ros vs. RHRSP/veh, *P* = 0.0019. ^∗^RHRSP/veh vs. sham-operated rats, *P* < 0.05. ^#^RHRSP/ros vs. RHRSP/veh, *P* < 0.05. *n* = 19 in RHRSP/veh and RHRSP/ros; *n* = 11 in the sham-operated group.

As shown in Figure 3(a), Luxol fast blue staining showed obvious WML in vehicle-treated RHRSPs. Disarrangement of nerve fibers, obvious vacuoles, disappearance of myelin, and nerve fibers were observed in the corpus callous. Nerve fibers were neat in sham-operated rats. The severity of WML was ameliorated by rosuvastatin administration. There was a significant difference between the vehicle-treated RHRSPs and rosuvastatin-treated RHRSPs in the severity of WML as evaluated by WML grades (RHRSP/veh vs. sham-operated, *P* = 0.0027; RHRSP/ros vs. RHRSP/veh, *P* = 0.0019) (Figure 3(b)).

### 3.4. Rosuvastatin Reduced the Loss of Tight Junction Proteins of BBB in the Corpus Callosum

**Figure 4:**
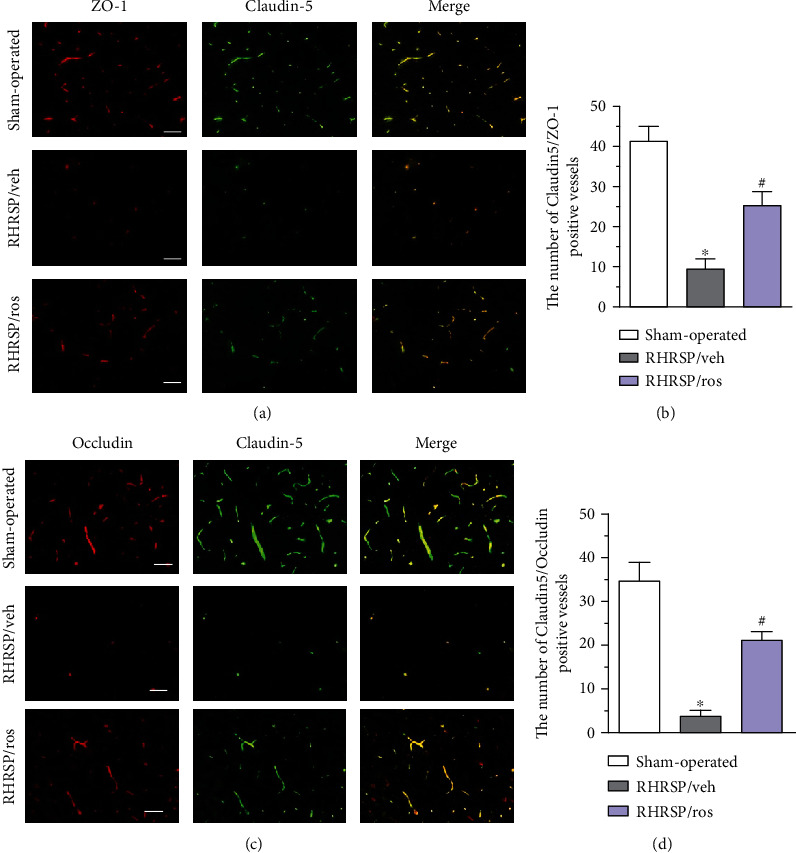
Rosuvastatin reduced the loss of tight junction proteins of blood-brain barrier in corpus callosum. (a) Representative images of vessels marked by ZO-1 (red) and Claudin-5 (green). Scale bar: 50 *μ*m. (b) Quantitative analysis of the number of Claudin-5/ZO-1-positive vessels. (c) Representative images of vessels marked by Occludin (red) and Claudin-5 (green). Scale bar: 50 *μ*m. (d) Quantitative analysis of the number of Claudin-5/Occludin-positive vessels. ^∗^RHRSP/veh vs. sham-operated rats, *P* < 0.001. ^#^RHRSP/ros vs. RHRSP/veh, *P* < 0.001. *n* = 19 in RHRSP/veh and RHRSP/ros; *n* = 11 in the sham-operated group.

The expression of ZO-1, Occludin, and Claudin-5 in the corpus callosum was detected by immunofluorescence. As shown in Figures 4(a) and 4(b), strong fluorescence for vessels marked by ZO-1 and Claudin-5 of sham-operated rats was significantly decreased in the RHRSP/veh group, and the expression of these proteins was significantly upregulated in the rosuvastatin-treated RHRSPs. Vessels marked by Claudin-5 (green) and Occludin (red) in the corpus callosum showed a similar tendency (Figures 4(c) and 4(d)). Immunofluorescence analysis revealed that ZO-1, Occludin, and Claudin-5 exhibited a continuous distribution along vessels border in sham-operated rats and rosuvastatin-treated RHRSPs. However, discontinuous distribution at tight junctions was observed in the vehicle-treated RHRSPs.

### 3.5. Rosuvastatin Reduced A*β* Deposits in the Cortex and Hippocampus

**Figure 5:**
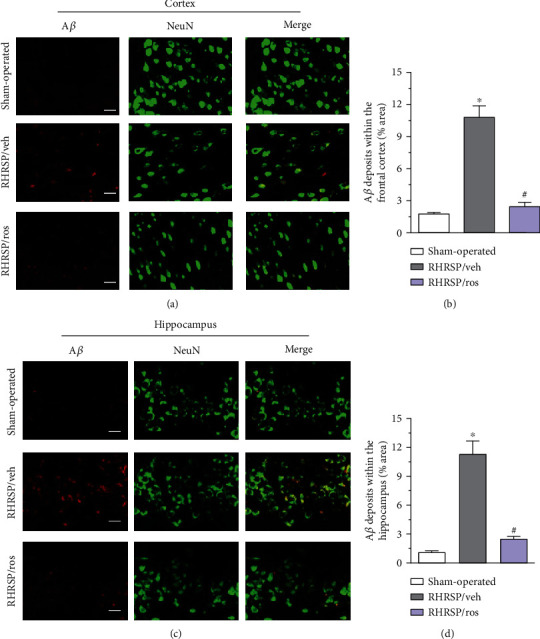
Rosuvastatin ameliorated A*β* deposits in the cortex and hippocampus. (a) Representative images of A*β* protein accumulation in the frontal cortex. Scale bar: 25 mm. (b) Quantitative analysis of Ab deposits in the frontal cortex. (c) Representative images of A*β* protein accumulation in the hippocampus. Neurons were stained with NeuN (green). A*β* was stained with A*β*3-16 (red). Scale bar: 25 mm. (d) Quantitative analysis of A*β* deposits in the hippocampus. ^∗^RHRSP/veh vs. sham-operated rats, *P* < 0.001. ^#^RHRSP/ros vs. RHRSP/veh, *P* < 0.001. *n* = 19 in RHRSP/veh and RHRSP/ros; *n* = 11 in the sham-operated group.

The presence of A*β* deposits in the frontal cortex and hippocampus was investigated. A*β* immunoreactivity (red) in the frontal cortex of the sham-operated rats was apparently increased in the RHRSP/veh group (Figures 5(a) and 5(b)). The increase in A*β* immunoreactivity was attenuated in the RHRSP/ros group. Neurons in the cortex were stained with NeuN (green), and most of the A*β* fluorescence merged with the NeuN immunoreactivity, indicating intracellular localization of A*β*.

Accumulations of A*β* were also observed in the CA1 region of the hippocampus in the RHRSP/veh group (Figures 5(c) and 5(d)). Similar to that observed in the frontal cortex, enhanced A*β* immunoreactivity was markedly attenuated by rosuvastatin administration. Again, the presence of intracellular accumulation of A*β* in pyramidal neurons was detected using double staining for A*β* and NeuN.

## 4. Discussion

In our current study, the data indicated that benefits on the cognitive function in RHRSPs with rosuvastatin were mediated by ameliorating WMLs and alleviating A*β* deposits.

The effects of statins on blood pressure remain controversial. A large cohort study has suggested that the statin therapy is associated with significantly lower diastolic blood pressure [17]. Another clinical research has also indicated that rosuvastatin has an additive antihypertensive effect in patients with poorly controlled hypertension [18]. However, animal studies suggested that rosuvastatin failed to reduce blood pressure in rats with hypertension [19, 20]. In the present study, rosuvastatin at a dose of 10 mg/kg failed to lower the blood pressure levels in RHRSPs. The discrepancy may be explained by the difference in disease, dose selection, or treatment duration. Moreover, the consistency of blood pressure in RHRSP/veh and RHRSP/ros groups suggested that rosuvastatin prevented cerebrovascular injury and cognitive decline independent of blood pressure.

It remains controversial whether statins have beneficial effects on cognitive impairment of Alzheimer's disease (AD) and/or vascular dementia. In animal studies, the administration of statins in early stage of disease improved the cognitive function of adult APP mice (6 months) [21], partly due to the improvement of cerebrovascular reactivity, whereas such positive effect on cognitive impairment was not observed in aged AD mice (9-12 months) [21, 22]. Two randomized control trials, the Heart Protection Study and the Prospective Study of Pravastatin in the Elderly at Risk study, observed no positive effect of statins on vascular dementia [21, 22]. In a further systemic review, statin therapy in the elderly seemed to fail to protect cognitive function [23]. These theories were not substantiated in our study. On the contrary, we found that rosuvastatin exerted beneficial roles in cognitive impairment of long-term hypertensive rats. We postulate that cognitive decline resulting from high blood pressure is not only associated with the cortex and hippocampus, but also with WML [24, 25]. The differences of pathogenesis between hypertension and other diseases may account for the disparity in conclusions. Since the grouping criteria of those randomly controlled trials mainly focused on dyslipidemia, there lacked further discussion about the effects of statins on the cognitive function of patients with only hypertension.

Our study found that rosuvastatin reduced the progression of WML in hypertensive rats by ameliorating the disarrangement of nerve fibers, obvious vacuoles, and demyelination in corpus callous. The effects of statins on WML remain to be further investigated. Several clinical researches showed that compared to the placebo group, statin use reduced the progression of WML [26–28]. The Regression of Cerebral Artery Stenosis study suggested that the beneficial effects of statins on WML may be independent to their lipid-lowering effects [26]. However, these clinical studies failed to clarify potential mechanisms of statins on WML progression. Evidences provided by our previous works showed that WML was induced by the disruption of tight junction proteins of BBB caused by long-term hypertension [29]. Our present study showed that rosuvastatin attenuated the loss of tight junction proteins such as ZO-1, Occludin, and Claudin-5. Therefore, we supposed that rosuvastatin attenuated WML partly by reducing the loss of tight junction proteins. These results were consistent with previous findings, which showed the protective effects on BBB integrity in a hypertensive rat model induced by L-N*^*ω*^*-nitro-l-arginine methyl ester hypertension and angiotensin II [30].

Furthermore, we observed lower A*β* plaque load in rosuvastatin-treated hypertensive rats, in consistent with part of animal studies in AD mice [22, 31, 32]. Statin could alleviate A*β* deposits by modulating APP maturation and phosphorylation [33], increasing A*β* clearance [31], upregulating the expression of acetylcholine receptors [34], and other potential mechanisms. Meanwhile, most cross-sectional studies demonstrate a significant association between WML and A*β* burden [9, 35], indicating that the reduction of WML may alleviate the accumulation of A*β*. Although some studies identify no correlation between WML and A*β* deposits [36], these results could be explained by the various criteria of WML [9]. Interstitial fluid accumulated in the extracellular spaces in WML and gradually caused impaired perivascular drainage of the brain [37], thereby resulting in lower A*β* clearance. Therefore, we supposed that the attenuation of WML partly contributed to the reduction of A*β* plaque load in hypertension rats. As A*β* deposits could cause synapse dysfunction [38] and neurodegeneration, our present data indicated that the decrease of A*β* burden by rosuvastatin, partly due to the attenuation of WML, contributed to the recovery of cognitive function.

## 5. Conclusions

The present study showed that rosuvastatin could improve the cognitive function of long-term hypertensive rats partly by attenuating WML and A*β* burden. The protective effects of rosuvastatin on BBB integrity might partly contribute to the reduction of WML.

## Data Availability

The datasets used and analyzed during the current study are available from the corresponding author on reasonable request.
